# The excess insulin requirement in severe COVID‐19 compared to non‐COVID‐19 viral pneumonitis is related to the severity of respiratory failure and pre‐existing diabetes

**DOI:** 10.1002/edm2.228

**Published:** 2021-02-11

**Authors:** Sam M. Lockhart, Harry Griffiths, Bogdan Petrisor, Ammara Usman, Julia Calvo‐Latorre, Laura Heales, Vishakha Bansiya, Razeen Mahroof, Andrew Conway Morris

**Affiliations:** ^1^ MRC Metabolic Diseases Unit Wellcome Trust‐Medical Research Council Institute of Metabolic Science University of Cambridge Cambridge UK; ^2^ Wolfson Diabetes and Endocrinology Clinic Cambridge University Hospital NHS Foundation Trust Cambridge UK; ^3^ John Farman Intensive Care Unit Addenbrooke’s Hospital Cambridge UK; ^4^ University Division of Anaesthesia Department of Medicine University of Cambridge Cambridge UK

**Keywords:** COVID‐19, diabetes, insulin resistance, stress hyperglycaemia

## Abstract

**Introduction:**

Severe COVID‐19 has been anecdotally associated with high insulin requirements. It has been proposed that this may be driven by a direct diabetogenic effect of the virus that is unique to SARS‐CoV‐2, but evidence to support this is limited. To explore this, we compared insulin requirements in patients with severe COVID‐19 and non‐COVID‐19 viral pneumonitis.

**Methods:**

This is a retrospective cohort study of patients with severe COVID‐19 admitted to our intensive care unit between March and June 2020. A historical control cohort of non‐COVID‐19 viral pneumonitis patients was identified from routinely collected audit data.

**Results:**

Insulin requirements were similar in patients with COVID‐19 and non‐COVID‐19 viral pneumonitis after adjustment for pre‐existing diabetes and severity of respiratory failure.

**Conclusions:**

In this single‐centre study, we could not find evidence of a unique diabetogenic effect of COVID‐19. We suggest that high insulin requirements in this disease relate to its propensity to cause severe respiratory failure in patients with pre‐existing metabolic disease.

## Graphical abstract

Our observational study demonstrates that insulin requirements in patients with severe COVID‐19 are associated with severity of respiratory failure and pre‐existing diabetes. When these features were accounted for, there was no difference in insulin requirements in patients with COVID‐19 or non‐COVID‐19 viral pneumonitis.

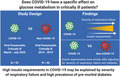

## 1 INTRODUCTION

There is emerging interest in the effect of COVID‐19 on glucose homeostasis, with some suggesting that COVID‐19 may be a uniquely diabetogenic virus.^1^ Observations advanced to support this hypothesis include anecdotal reports of high insulin requirements in patients with severe COVID‐19.^2^, ^3^, ^4^ However, to date there has been no longitudinal study of patients with COVID‐19 to empirically assess insulin requirements. We sought to address this deficiency by comparing insulin requirements in patients admitted to intensive care with severe COVID‐19 to a historical control group with severe non‐COVID‐19 viral pneumonitis.

## 2 RESEARCH DESIGN AND METHODS

### 2.1 Case identification

Patients admitted to ICU in Addenbrooke's Hospital, Cambridge, with a positive SARS‐CoV‐2 test were identified. Patients were deemed to have COVID‐19 pneumonitis and included in the study if they were SARS‐CoV‐2 positive and were treated for respiratory failure that was attributed to COVID‐19 pneumonitis by the treating team during their ICU stay.

The non‐COVID‐19 viral pneumonitis cohort was identified from the ICU’s routinely collected audit data from 2014 to 2019. Patients admitted with a diagnosis of viral pneumonia recorded as their primary, secondary or ultimate reason for admission to intensive care were included in the analysis.

All eligible participants were included.

### 2.2 Data collection

Data collection was undertaken manually from electronic medical records by trained, clinical investigators. Blood glucose data were collected using proprietary analytics software, QlikView. Further detail is available in Appendix S1.

### 2.3 Matching

Patients admitted with COVID‐19 pneumonitis were matched according to diabetes status and level of respiratory support received in a 1:1 ratio to a historical control cohort of patients admitted to ICU with non‐COVID‐19 viral pneumonitis. Further data are available in Appendix S1.

### 2.4 Statistical analysis

Details of the statistical analysis are available in Appendix S1.

## 3 RESULTS

During the study period March to June 2020, we identified 92 patients treated for severe COVID‐19 within our intensive care unit (Appendix S1: Figure S1). In univariate linear regression analysis, BMI, pre‐existing type 1 or type 2 diabetes and severity of respiratory failure assessed by ordinal scale (0—self ventilating, 1—mechanical ventilation, 2—neuromuscular blockade, 3—nebulized epoprostenol, 4—prone ventilation, 5—extracorporeal membrane oxygenation) were all associated with insulin dose when analysed by the average dose across the whole ICU stay or by the highest cumulative insulin dose over a 24‐h period (Appendix S1: Table S1).

**FIGURE 1:**
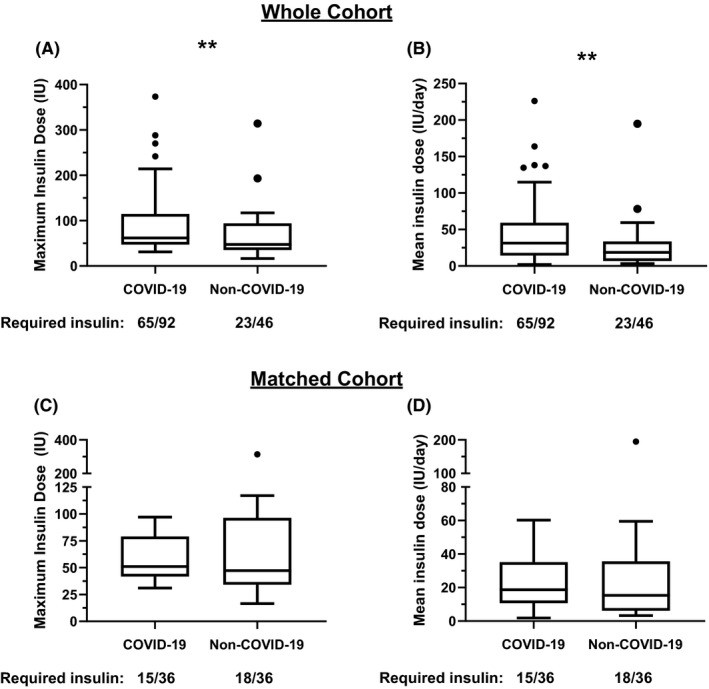
Insulin requirements of patients admitted to ICU with COVID‐19 and non‐COVID‐19 viral pneumonitis. (A) Tukey box plots of maximum cumulative insulin requirements in a single day in the complete cohort, *p* = .003 by Mann‐Whitney *U* test. (B) Tukey box plots of mean daily insulin dose day in the complete cohort, *p* = .003 by Mann‐Whitney *U* test. (C) Tukey box plots of maximum cumulative insulin requirements in a single day in the matched cohort, *p* = .46 by Wilcoxon signed rank test. (D) Tukey box plots of mean daily insulin dose day in the matched cohort, *p* = .52 by Wilcoxon signed rank test. For clarity only insulin dose for patients receiving insulin plotted (ie no zero values plotted) but statistical tests are performed on the whole dataset. IU, international units

To determine if the observed insulin requirements were a unique feature of COVID‐19, we examined insulin requirements in 46 patients admitted to our ICU with a diagnosis of non‐COVID‐19 viral pneumonitis between 2014 and 2019. 89% of the non‐COVID‐19 cohort had a confirmed viral isolate consistent with viral pneumonia. Five patients included in the analysis did not have any relevant positive virology related to their ICU admission but were diagnosed with viral pneumonitis on clinical grounds while two patients had more than 1 virus identified that could have explained their symptoms (Appendix S1: Table S2 for a breakdown of viral isolates). Insulin requirements were higher in patients with COVID‐19 versus non‐COVID‐19 viral pneumonitis while mean glycaemic indices were comparable in both groups (Figure 1A,B and Appendix S1: Figure S2A,B). However, the groups were poorly balanced with respect to diabetes status and requirement for respiratory salvage therapies (Appendix S1: Table S3), and multiple regression analysis did not identify any association between COVID‐19 and insulin dose after adjustment for pre‐existing diabetes and severity of respiratory failure (Table 1). While severity of respiratory failure was independently associated with insulin requirement, we did not find a similar relationship for vasopressor use or APACHE II score (data not shown). When COVID‐19 and non‐COVID‐19 viral pneumonitis patients were matched (1:1, *N* = 36 per group) by the level of respiratory support required and pre‐existing diabetes status, the proportion of patients requiring insulin (COVID‐19: 41%, non‐COVID‐19: 49%) and insulin dose was similar in those who were treated with insulin (Figure 1C,D and Appendix S1: Figure S2C,D). We acknowledge that diabetes is a heterogenous condition with different intensity of treatment and adequacy of glycaemic control and controlling for it as a single variable is limited. However, the number of patients with type 1 diabetes in COVID‐19 and non‐COVID‐19 was the same and the treatments used in type 2 diabetes COVID‐19 and non‐COVID‐19 viral pneumonitis are not dissimilar (Appendix S1: Table S4). Unfortunately, a large proportion of patients in our study did not have pre‐morbid HbA_1c_ values on records at our institution therefore precluding meaningful comparison of pre‐morbid glycaemic control.

**TABLE 1:** Correlation coefficients ± standard error, multiple *R*‐squared and *p*‐values from multivariable linear regression analysis of maximum insulin requirement in a single day and mean insulin requirements per day, following a square root transformation

	Beta	*p*‐Value
Maximum insulin requirement in a single day, *R* ^2^ = .47		
COVID‐19	0.10 ± 0.71	.89
Diabetes mellitus	4.66 ± 0.75	<.001
Respiratory support	1.41 ± 0.21	<.001
Mean insulin requirements per day, *R* ^2^ = .50		
COVID‐19	0.20 ± 0.50	.71
Diabetes mellitus	5.60 ± 1.05	<.001
Respiratory support	1.64 ± 0.26	<.001

Finally, we sought to determine if the insulin requirements observed in patients with COVID‐19 resolved in convalescence. Diabetes therapies used in patients with COVID‐19 and non‐COVID‐19 viral pneumonitis are summarized in Appendix S1: Table S4. We had information on discharge medication in 61/62 patients who survived to hospital discharge. Of these patients, 39 required insulin while in ICU but on discharge only five patients were discharged from hospital on subcutaneous insulin, four of whom were on insulin prior to admission. The one patient commenced on insulin had type 2 diabetes prior to admission managed with oral therapy, and insulin was discontinued soon after discharge when they were readmitted with hypoglycaemia. There was no initiation or escalation in oral hypoglycaemic agents in the cohort; however, we cannot comment on the adequacy of glycaemic control. Similar findings from a smaller cohort have recently been reported.^3^

## 4 DISCUSSION

We report here, to the best of our knowledge, the first longitudinal assessment of glycaemia and insulin requirements in patients who are critically ill with COVID‐19. Our data suggest that high insulin requirements observed in patients with severe COVID‐19 are related to the severity of respiratory failure and pre‐existing diabetes mellitus rather than a direct diabetogenic effect of SARS‐CoV‐2. Therefore, there is currently no rationale to adopt novel strategies or different glycaemic thresholds to manage hyperglycaemia in COVID‐19 compared to other viral pneumonias and several reviews have outlined practical approaches to the management of hyperglycaemia in COVID‐19.^2^, ^4^, ^5^ Moreover, the comparable insulin requirements in COVID‐19 and non‐COVID‐19 viral pneumonitis when severity of illness and pre‐existing diabetes are accounted for argue against a direct action of SARS‐CoV‐2 on the pancreatic β‐cell as the likely cause of hyperglycaemia in COVID‐19, as has been suggested previously.^6^ In addition, our data suggest that the hyperglycaemia observed in patients with COVID‐19 is transient and resolves in convalescence and leads us to caution studies describing new‐onset diabetes in patients with COVID‐19 based on cross‐sectional blood glucose measurements.^7^

Our findings are complementary to a recent analysis that examined time in target glucose range in patients admitted to ICU with COVID‐19. The authors found that ventilator use, neuromuscular blockade and use of high‐flow nasal oxygen all predicted poor glycaemic control.^8^ Interestingly, this study compared glycaemia and insulin requirements in 93 COVID‐19 patients to all (*N* = 469) contemporaneous ICU admissions (for any reason) without COVID‐19 and found that COVID‐19 patients had poorer glycaemic control and higher insulin requirements. However, the COVID‐19 group required much more respiratory support compared to the non‐COVID‐19 group and the authors did not make any attempt to adjust for this difference.

While preparing our paper for publication, two separate analyses have suggested that insulin therapy in hospital may be associated with increased mortality in patients with type 2 diabetes.^9^, ^10^ One of these reports has been widely interpreted as supporting the supposition that insulin therapy is detrimental in COVID‐19.^11^ We would take the view that these findings represent reverse causation—whereby those with severe illness require insulin to treat stress hyperglycaemia. An alternative explanation is that the optimal glycaemic targets in patients with type 2 diabetes and severe illness are unclear, and some observational analyses have suggested more liberal targets in patients with type 2 diabetes may be preferable—a hypothesis being examined by the LUCID Randomized Controlled Clinical Trial.^12^

It is interesting to speculate on the pathophysiological mechanisms linking severity of respiratory failure to insulin requirements in viral pneumonitis. It is well established that acute illness causes stress hyperglycaemia and the production of pro‐inflammatory cytokines and counterregulatory hormones are likely key players in this process.^13^ What is less well established is that severity of respiratory failure specifically is associated with insulin resistance and stress hyperglycaemia. Causative mechanisms likely overlap but some pathogenic processes may be specific to tissue dysoxia. Examining insulin requirements and glycaemia treated with liberal and conservative oxygen strategies in randomized controlled trials may be instructive in this regard.

Our analysis is limited by its relatively small sample size and its single‐centre nature. Further determination of the specific effects of COVID‐19 infection as opposed to the effects of general critical illness will require collection of data from larger, well‐matched cohorts with other precipitating causes of stress hyperglycaemia.

It should be noted that we cannot exclude the existence of rare hyperglycaemic syndromes triggered by SARS‐CoV‐2 infection. For example, our study cannot inform the aetiological role of SARS‐CoV‐2 infection in autoimmune diabetes as has been studied in larger epidemiological analyses.^14^, ^15^, ^16^ Large, registry‐based studies (eg COVIDIAB registry) with prolonged follow‐up will be required to study this and to determine the long‐term effects of COVID‐19 infection on glucose metabolism more broadly.

In conclusion, high insulin requirements in severe COVID‐19 likely relate to the severity of respiratory failure and the high prevalence of metabolic disease in patients with severe illness. When these factors are accounted for, insulin requirements are comparable to that seen in non‐COVID‐19 viral pneumonitis.

## CONFLICT OF INTERESTS

The authors have no relevant conflict of interests to declare.

## AUTHOR CONTRIBUTIONS

SL, VB, RM and ACM conceived and designed the study. SL, HG, BP, AU and LH collected the data. SL and ACM wrote the manuscript and performed the analysis. All authors reviewed the manuscript for important intellectual content. ACM is the guarantor of this work.

## Supporting information

Appendix S1

## Data Availability

This was a retrospective review of routinely collected clinical data that was approved by the responsible healthcare organization, and we do not have permission to make data publicly available.
