# Supplementary material for: The excess insulin requirement in severe COVID‐19 compared to non‐COVID‐19 viral pneumonitis is related to the severity of respiratory failure and pre‐existing diabetes
Source: Endocrinol Diabetes Metab. 2021 Feb 11;4(3):e00228. doi: 10.1002/edm2.228 (PMC7995054; doi:10.1002/edm2.228)
Supplement: Supplementary file 1 — Appendix S1 [file EDM2-4-e00228-s001.pdf]

Supplementary Appendix

The excess insulin requirement in severe COVID-19 compared to non-COVID-19 viral pneumonitis is related to the severity of respiratory failure and pre-existing diabetes.

Sam Lockhart, Harry Griffiths, Bogdan Petrisor, Ammara Usman, Julia Calvo-Latorre, Laura Heales, Vishakha Bansiya, Razeen Mahroof, Andrew Conway Morris

Methods

Study Centre

All patients included in the study were managed in Addenbrooke's hospital intensive care unit which includes a 20-bed general intensive care unit with a further 12 high and intermediate dependency beds which were repurposed as critical care beds as required during the pandemic, and a 27-bed specialist neurocritical care unit.

Glycemic control

Our departmental policy for management of hyperglycaemia in critical care advises commencement of a variable rate intravenous insulin therapy when two consecutive blood glucose readings are >10 mmol/L if there has been no recent (within 6 hours) hypoglycaemia. The rate of intravenous insulin infusion is adjusted by bedside nursing staff depending on blood glucose according to a standard prescription which can be titrated by medical staff with the aim of maintain a target blood glucose of 6-10mmol/L. In patients with persistent hyperglycaemia or high insulin requirements, subcutaneous basal insulin may be commenced under the advice of the specialist diabetes team. When this was added it was solely with the intention of improving glycaemic control.

There was not, to our knowledge, any case where subcutaneous insulin therapy was used in an effort to spare syringe pumps during the COVID-19 pandemic.

Data Collection

Demographic data was manually collected from review of the participant electronic medical record. Admission values for cross sectional variables such as age and BMI were recorded.

A diagnosis of pre-morbid diabetes mellitus was recorded if the patient was on any anti-hyperglycemic therapy, if they had a recorded diagnosis of diabetes mellitus in their problem list or if they had a pre-morbid HbA_{1c} that was diagnostic of Diabetes according to World Health Organisation Diagnostic Criteria (48 mmol/mol or over). Diabetes mellitus was attributed to Type 2 Diabetes unless there was a positive diagnosis of Type 1 Diabetes recorded in the notes. Prior to admission hyperglycaemic therapy was documented according to the reconciled medications list in the electronic medical record and the medicines administration record was used to determine anti-hyperglycaemic therapies used while in hospital. The discharge summary was used to determine antihyperglycaemic medications that were continued on discharge. Insulin doses recorded were the sum of all subcutaneous and intravenous insulin administered during the patient’s stay in intensive care.

Acute corticosteroid use was examined and the number of days that corticosteroids were administered was recorded. We did not include corticosteroid use that was prescribed regularly prior to admission.

APACHE II scores were collected on admission to intensive care and were obtained from routinely collected audit data.

Age, Sex, BMI (on ICU admission), obesity status and level of respiratory support were manually collected from the patient's electronic medical record by trained investigators.

Follow-up

For glycaemia and insulin requirements, participants were followed up until they were discharged from ICU or died. Length of ICU stay is detailed in Supplementary Table 3. As noted in the main manuscript we had information on discharge medication on 61/62 patients who survived to hospital discharge, the one patient we did not have information on was repatriated to their local hospital after completion of their ICU stay.

Matching

Patients admitted with COVID-19 pneumonitis were matched in a 1:1 ratio to a historical control cohort of patients admitted to ICU with non-COVID-19 viral pneumonitis. Subjects were strictly matched so that the level of respiratory support received (e.g. mechanical ventilation, neuromuscular blockade, epoprostenol therapy, prone ventilation and extra-corporeal membrane oxygenation) and diabetes status were identical. If there was more than one suitable match then age was used (as age was significantly associated with insulin requirements when added to the model presented in supplementary table 3), if age was similar (tolerance ± 5 years) then BMI was reviewed, if BMI was similar (tolerance ±2.5 kg/m^2) then Sex was reviewed. These criteria were pre-specified prior to matching and undertaken blinded to knowledge of insulin requirements.

Statistical analysis

Continuous variables were assessed for normality by visual inspection of histograms of each variable and performing the Shapiro-Wilk's Test. Student's t-test was used for comparing means of normally distributed data, Mann-Whitney U test and Wilcoxon Rank test were used for comparing unpaired and paired non-normally distributed data, respectively. All P-Values reported are from two-tailed tests.

The level of maximum respiratory support required during the ICU stay was assigned an ordinal scale : 0 – self ventilating, 1- mechanical ventilation, 2 – neuromuscular blockade, 3 – nebulized epoprostenol, 4 – prone ventilation, 5 – extracorporeal membrane oxygenation. The regression co-efficients and p-values reported are from a model encoding respiratory support as a continuous variable but a sensitivity analysis encoding respiratory support as a categorical variable showed similar results. For the purposes of analysis, the number of days receiving steroids was normalized according to the length of stay to avoid the confounding effects of duration of ICU stay. Insulin requirements (both maximum use in a single day and mean daily use) were right skewed and were squareroot transformed to facilitate regression analysis. For multiple regression analysis diabetes status and level of required respiratory support were included in the model as these two variables were unbalanced in the COVID-19 and non-COVID-19 viral pneumonitis groups and were the most significant predictors of insulin requirement. The addition of age and BMI to the model (which were both significant in univariate analysis) did not improve the predictive ability of the model as assessed by a likelihood ratio test, nor did they alter the relationship between COVID-19 and insulin requirement.

The results reported are from linear regression analysis of the whole data set but similar results were observed (e.g. COVID-19 was not a significant predictor of insulin requirements after addition of an index of respiratory support and diabetes status were added to the model) using tobit regression with censoring at 0 or if insulin requirement was dichotomized to those requiring and not requiring insulin and analyzed by binomial regression.

Statistical analysis was conducted using Graphpad Prism version 8.4.3 and R.

Clinical Governance and Oversight

As a retrospective analysis of anonymized, routinely collected data the requirement for ethical committee review and consent was waived, the evaluation was registered with the responsible healthcare organization (Cambridge University Hospitals NHS Foundation Trust).

Supplementary Appendix Figure 1

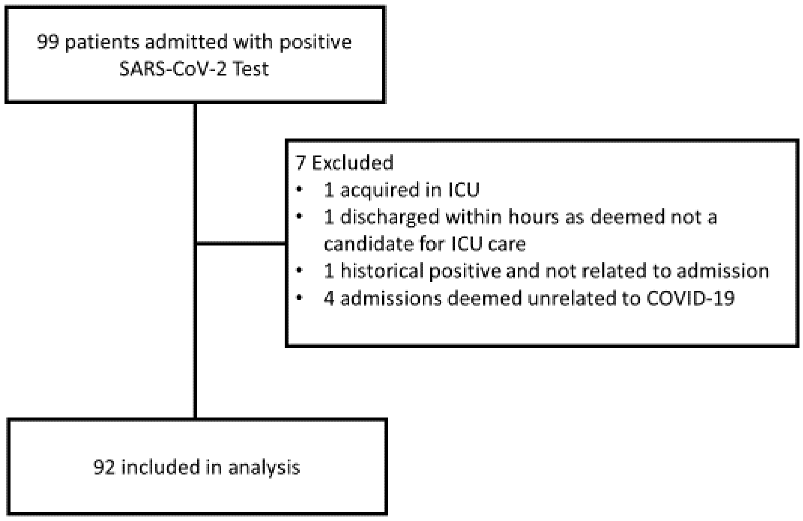

Supplementary Figure 1 – Consort diagram illustrating patient inclusion

Supplementary Appendix Figure 2

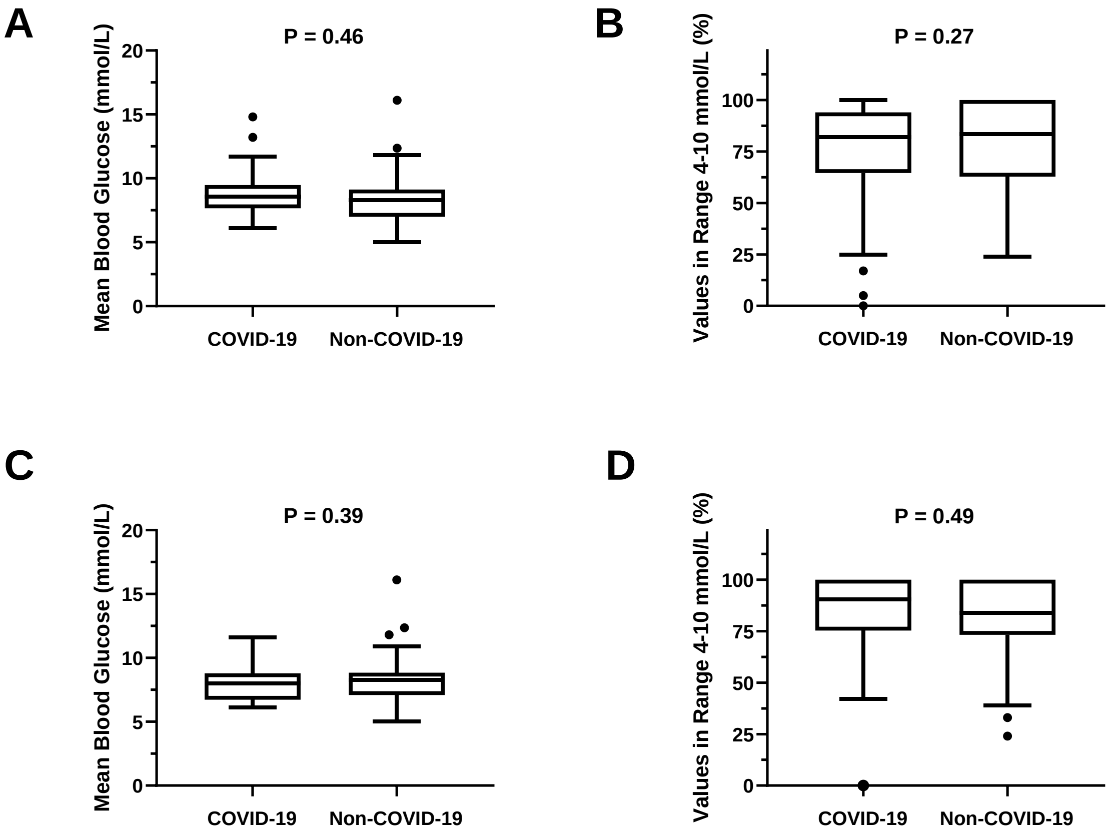

Supplementary Figure 2 – **Glucose levels of patients of patients admitted to ICU with COVID-19 and non-COVID-19 viral pneumonitis. A)** Tukey Box plots of mean blood glucose in the complete cohort, P = 0.42 by t-test **B)** Tukey Box plots of percentage time in target range (4-10mmol/L) in the complete cohort, P = 0.27 by Mann-Whitney U test **C)** Tukey Box plots of mean blood glucose in the matched cohort, P = 0.39 by paired t-test. **D)** Tukey Box plots of percentage time in target range (4-10mmol/L) in the matched cohort, P = 0.49 by Wilcoxon signed rank test

Supplementary Appendix Table 1

	**Beta**	**R^2**	**P-Value**
**Maximum insulin requirement in a single day**			
**Age**	0.08 ± 0.04	0.05	0.03
**Male**	-0.23 ±1.10	0.0004	0.83
**Type 1 Diabetes**	7.58 ± 3.42	0.04	0.03
**Type 2 Diabetes**	5.60 ± 1.05	0.23	<0.001
**BMI**	0.19 ± 0.08	0.06	0.02
**APACHE II**	0.19 ± 0.09	0.04	0.05
**Respiratory Support**	1.64 ± 0.26	0.30	<0.001
**Steroid Exposure**	1.37 ± 1.80	0.006	0.45
**Mean insulin requirements per day**			
**Age**	0.04 ± 0.03	0.02	0.14
**Male**	-0.32 ± 0.82	0.02	0.70
**Type 1 Diabetes**	6.34 ± 2.52	0.07	0.01
**Type 2 Diabetes**	4.53 ± 0.75	0.29	<0.001
**BMI**	0.16 ± 0.06	0.08	0.008
**APACHE II**	0.12 ± 0.07	0.03	0.08
**Respiratory Support**	1.10 ± 0.20	0.25	<0.001
**Steroid Exposure**	1.33 ± 1.33	0.01	0.32

Supplementary Table 1 – Correlation coefficients ± standard error, multiple R-squared and p-values from univariate linear regression analysis of maximum insulin requirement in a single day and mean insulin requirements per day, following a square root transformation. BMI – Body Mass Index, APACHE II - Acute Physiology And Chronic Health Evaluation II.

Supplementary Appendix Table 2

Viral Isolate	N
**Influenza A**	21
**Influenza B**	4
**Influenza H1N1**	1
**Enterovirus**	2
**Varicella Zoster Virus**	1
**Cytomegalovirus**	1
**Human Metapneumavirus**	2
**Rhinovirus**	3
**Parainfluenza**	6
**Non-Typeble picornavirus**	1
**Adenovirus**	1
**No viral isolate – clinical diagnosis**	5

Supplementary Table 2 – Viral isolates in the Non-COVID-19 viral pneumonitis cohort.

Supplementary Appendix Table 3

	COVID-19 (N=92)	Non-COVID-19 (N=46)	Matched COVID-19 (N=36)	Matched Non-COVID-19 (N=36)
**Age**	62 (50, 69)	62 (48.5, 68)	62 (49, 67.5)	62.5 (57, 68.25)
**Male**	68% (63)	57% (56)	61.1% (22)	61.1% (22)
**Type 1 Diabetes**	2% (2)	4% (2)	-	-
**Type 2 Diabetes**	24% (22)	11% (5)	8.3% (3)	8.3% (3)
**BMI**	28.5 (25.8, 32.4)	27.2 (24.7, 32.3)	28.5 (25.8, 31.2)	27.5 (25.8, 32.3)
**APACHE II**	15 (12, 19)	17 (13.5, 20.5)	13.5 (11, 18)	16 (13.25, 20)
**Obesity**	39% (36)	26% (12)	35.1% (13)	27.8% (10)
**Mechanical Ventilation**	83% (76)	67% (31)	61.1% (22)	61.1% (22)
**Neuromuscular Blockade**	72% (66)	33% (15)	36.1% (13)	33.3% (12)
**Epoprostenol**	51% (47)	15% (7)	19.4% (7)	19.4% (7)
**Proned**	45.6% (42)	6.5% (3)	11.1% (4)	8.3% (3)
**ECMO**	7% (6)	2% (1)	2.8% (1)	2.8% (1)
**Steroids**	51.1% (47)	57.9% (29)	47.2% (17)	61.1% (22)
**ICU Survival**	72.8% (67)	71.1% (32)	80.6% (28)	69.4% (25)
**Length of Stay (Days)**	13 (7, 20)	5.5 (3, 14.75)	7 (4, 13)	5 (3, 14)

Supplementary Table 3 – Demographics of patients with COVID-19 viral pneumonitis and non-COVID-19 viral pneumonitis in the whole cohort and in the cohort matched for diabetes status and severity of respiratory failure. Categorical variables are summarized as % (n) and continuous variables as median (Q1, Q3). BMI – Body Mass Index, APACHE II - Acute Physiology And Chronic Health Evaluation II, ICU – Intensive Care Unit, ECMO – Extracorporeal Membrane Oxygenation.

Supplementary Appendix Table 4

	Metformin	SU	DPP4	GLP1RA	SGLT2i	TZD	Insulin
**COVID-19**	68.1% (15)	27.2% (6)	9.0% (2)	4.5% (1)	1 (4.5%)	0	36.4% (8)
**Non-COVID-19**	80% (4)	20% (1)	0	0	0	0	40% (2)

Supplementary Table 4 – Prior to admission diabetes therapies in patients with COVID-19 and Non-COVID-19 viral pneumonitis (Non-COVID-19) as a percentage (n) of all patients with type 2 diabetes in each group. SU – Sulfonylurea, DPP4 – Dipeptidyl peptidase-4, GLP1RA – Glucagon-Like Peptide-1 Receptor Analogue, SGLT2i – Sodium Glucose Like Transporter-2 inhibitor, TZD – thiazolidinediones.
